# The retina of the lab rat: focus on retinal ganglion cells and photoreceptors

**DOI:** 10.3389/fnana.2022.994890

**Published:** 2022-09-23

**Authors:** Caridad Galindo-Romero, María Norte-Muñoz, Alejandro Gallego-Ortega, Kristy T. Rodríguez-Ramírez, Fernando Lucas-Ruiz, María Josefa González-Riquelme, Manuel Vidal-Sanz, Marta Agudo-Barriuso

**Affiliations:** Experimental Ophthalmology Group, Instituto Murciano de Investigación Biosanitaria Virgen de la Arrixaca (IMIB-Arrixaca) & Ophthalmology Department, Universidad de Murcia, Murcia, Spain

**Keywords:** retinal ganglion cells, melanopsin, photoreceptors, topography, electroretinogram

## Abstract

Albino and pigmented rat strains are widely used in models to study retinal degeneration and to test new therapies. Here, we have summarized the main topographical and functional characteristics of the rat retina focussing on photoreceptors and retinal ganglion cells (RGCs), the beginning and end of the retinal circuitry, respectively. These neurons are very sensitive to injury and disease, and thus knowing their normal number, topography, and function is essential to accurately investigate on neuronal survival and protection.

## Introduction

Preclinical research in visual sciences is generally conducted in small mammals, mostly rodents and, to a lesser extent, rabbits or pigs. Rabbits are (usually) used to study the anterior part of the eye, the cornea, while rodents are (usually) used to investigate the retina, optic nerve, and retinorecipient areas. Within rodents, rats (*Rattus norvergicus*) and mice (*Mus musculus*) are the preferred species (>70,000 published articles in the last 20 years). Because they are nocturnal animals for specific research questions (i.e., circadian rhythms), diurnal rodents such as squirrels, Nile rats or common degus are also established models.

The layered structure of the retina is highly conserved along vertebrates (Ramón y Cajal, 2021). However, there are some cellular and regional differences, species-specific consequence of environmental and behavioral factors. For instance, rodents are nocturnal and thus their retina is rod-dominated. Most of primates and humans are diurnal, and though rods outnumber cones in the peripheral retina, they are almost absent in the central retina or macula. For color vision, like most mammals, rodents have two types of cones, S-cones (short light wavelength detecting-cones, or blue cones) and L/M-cones (medium-long light wavelength detecting-cones, red-green cones) while primates have three, S-, M-, and L- cones.

Primates are highly social animals and to correctly gauge the mood of their congeners, their vision must have high resolution. Thus, humans and primates have a specialized retinal area, the macula, with several rows of retinal ganglion cells (RGCs) and an RGC:cone ratio close to 1:1 characteristics that provide high resolution. Within the macula there is a depression, the fovea centralis, where most of retinal layers are missing but the cones, thus optimizing visual acuity. In rats there is no macula or fovea, instead they have a visual streak with higher RGC and L/M-cone densities (Salinas-Navarro et al., 2009; Ortín-Martínez et al., 2010).

Despite these differences, rats and mice are still the model of choice because they offer several advantages. They are small and adaptable, reproduce quickly and are easy to house and handle. In fact, both species have been used for research since the beginning of the 20th century, when the strains most commonly used today were created. Wistar rats (outbred, albino) were developed in 1906, Sprague Dawley (outbred, albino) in 1925, and C57/Bl6 mice (inbred pigmented) in 1921. These strains are now very well characterized genetically and physiologically (Hedrich and Bullock, 2012; Boorman, 2018). Furthermore, there are strains that spontaneously develop diseases such as diabetes, retinitis pigmentosa or glaucoma. In mice, genetic manipulation allows the generation of mutant or transgenic strains that, in many cases, are created to accurately mimic human diseases and pathologies. Genetic engineering is also used in rats but not very frequently.

Even though lack of melanin causes an impaired visual acuity, lower number of ipsilaterally projecting RGCs, defects in the optokinetic nystagmus, and a lower functional response (Lund, 1965; Balkema et al., 1981; Lund, 1986; Dräger and Balkema, 1987; Balkema and Drager, 1991; Prusky et al., 2002; Alarcón-Martínez et al., 2009; Nadal-Nicolás et al., 2012), albino rats are as used as pigmented ones in basic research.

Most retinal diseases proceed with the degeneration of RGCs or photoreceptors. The loss of these neurons may be caused by systemic dysfunctions such as diabetes or stroke, or congenital defects such as retinitis pigmentosa or Leber hereditary optic nerve neuropathy. There are other pathologies of unknown or complex etiology further complicated with risk factors such as glaucoma or age-related macular degeneration. To mimic these diseases there are many rat and mouse models that are either induced, spontaneous or genetically engineered (Shareef et al., 1995; Avilés-Trigueros et al., 2003; Pérez de Lara et al., 2014; Morgan and Tribble, 2015; Urcola et al., 2016; Mrejen et al., 2017; Sánchez-Migallón et al., 2018; Marchesi et al., 2021; Karademir et al., 2022; Subramaniam et al., 2022).

Consequently, the most studied retinal neurons are the photoreceptors and RGCs the beginning and end, respectively, of the neuronal retinal circuitry. Photoreceptors, as their name states, sense the light and transform it into action potentials that finally reach the RGCs which, in turn, send the processed information to the brain where ultimately the vision and non-vision light-induced responses occur.

Here, we compile for the first time the retinal topography and exact numbers of RGCs (vision and non-vision forming) and cone photoreceptors (L and S) and the electroretinographic waves used to study their function in the healthy rat retina.

## Retinal Ganglion Cells

Majority of RGCs are found in the ganglion cell layer (GCL), the innermost layer of the retina sharing location with displaced amacrine cells (50% of the cells in the GCL in rat) and glial cells (astrocytes and microglia, 10% of the cells in the GCL; Nadal-Nicolás et al., 2015, 2018). A small number of RGCs have their somas displaced to the inner plexiform layer (displaced RGCs; Nadal-Nicolás et al., 2014). Regardless of their location, RGCs are a heterogeneous population encompassing more than 40 subtypes according to their gene expression (Rheaume et al., 2018; Tran et al., 2019). Functionally, though, they are classified in two main groups: RGCs that convey vision-forming information and RGCs that send non-vision forming information.

Non-vision forming RGCs are intrinsically photosensitive (ipRGCs) because they express the photopigment melanopsin. Thanks to this pigment they sense light irradiance and regulate the circadian rhythm and the pupillary reflex (Provencio et al., 2000; Berg et al., 2019) ipRGCs, which encompass M1-M6 subtypes, have also some roles in pattern vision (Hattar et al., 2006; Ecker et al., 2010; Schmidt et al., 2011).

RGCs univocal identification is necessary to assess the extent of degeneration after a given insult or disease and to know if an experimental therapy is effective (Avilés-Trigueros et al., 2003; Vidal-Sanz et al., 2017; Sánchez-Migallón et al., 2018), and to do that most groups opt for immunodetection. There are several well-known RGC markers, i.e., proteins that in the retina are only expressed by RGCs. Among them Brn3a (Nadal-Nicolás et al., 2009) and RBPMS (Kwong et al., 2010; Rodriguez et al., 2014) stand out. Brn3a is expressed in vision-forming RGCs and thus it is a great tool to analyze vision and non-vision forming RGCs independently. RBPMS is expressed by all RGCs regardless of their function. Because melanopsin is a protein specifically expressed by ipRGCs, it’s immunodetection is the standard protocol to identify them. However, only M1-M3 ipRGCs express melanopsin at high enough levels for immunodetection. For this reason, ipRGCs identified with α-melanopsin antibodies are called melanopsin^+^ipRGCs (m^+^ipRGCs).

Using automated tools, our lab first described the total number and topographical distribution of Brn3a^+^ and m^+^ipRGCs quantified in retinal flatmounts imaged on the GCL (Nadal-Nicolás et al., 2009; Galindo-Romero et al., 2013), an approach now used by other groups (Geeraerts et al., 2016; Miesfeld et al., 2020; Masin et al., 2021). The total number of Brn3a^+^RGCs in albino rats (Sprague Dawley strain, SD) is 82,979 ± 1,787 and in pigmented (Pierald Viro Glaxo strain, PVG) a little higher, 84,818 ± 4,119. The m^+^RGC population is similar in both strains (2,047 ± 309 in SD and 2,098 ± 149 in PVG; Nadal-Nicolás et al., 2009; Galindo-Romero et al., 2013). Thus, m^+^ipRGCs represent ~2% of the RGCs.

**Figure 1:**
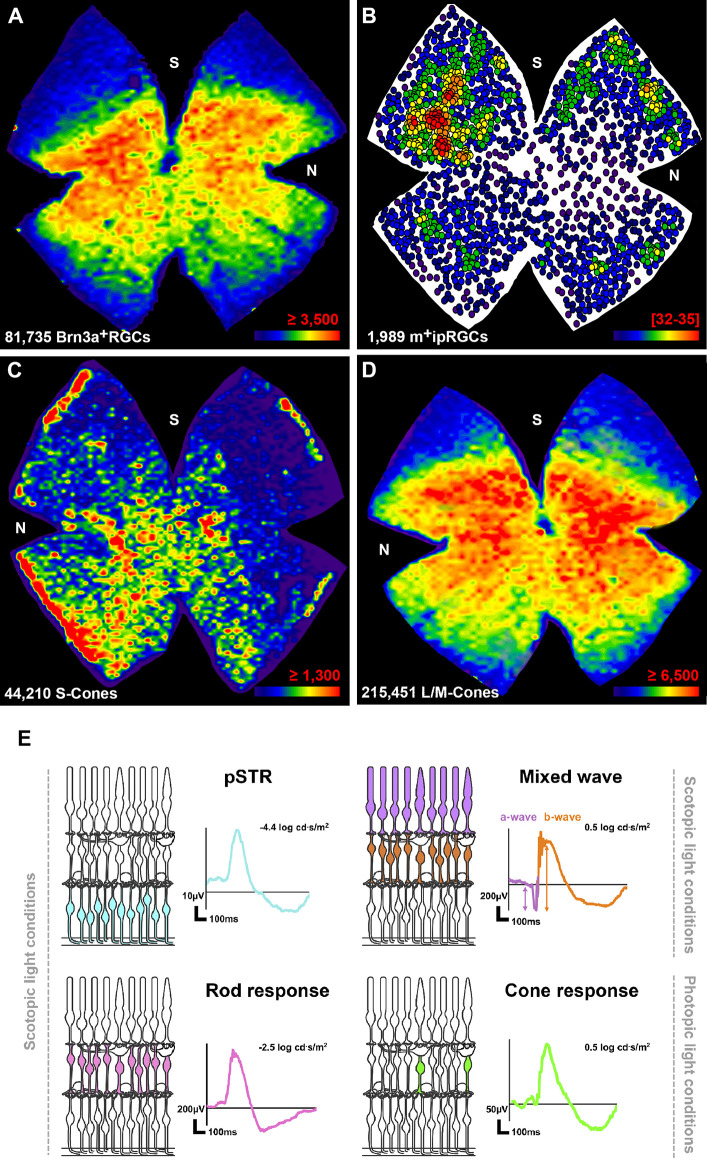
Anatomy and function of the healthy albino rat retina. **(A–D)** Distribution of RGCs and cone photoreceptors. Maps showing the topography of Brn3a^+^RGCs **(A)**, m^+^ipRGCs **(B)**, S-cones **(C)**, and L/M-cones **(D)** in the same retina from a 2 months old female SD albino rat. After flatmounting the retina each neuronal population was immunoidentified. Next the retina was imaged first vitreal side up (RGCs), and then flipped and imaged vitreal side down (photoreceptors). Then, each neuronal population was automatically quantified (total number shown at the bottom left of each map) and the topographical maps generated. For more details see references in text and below. **(A,C,D)** The distribution of Brn3a^+^RGCs and cone photoreceptors is visualized with isodensity maps that represent cell density in the retina with a color scale that goes from purple (0–500 cells/mm^2^) to red (≥3,500 Brn3a^+^RGCs/mm^2^, ≥1,300 S-cones/mm^2^, ≥6,500 L/M-cones/mm^2^). **(E)** The topography of m^+^ipRGCs is shown with a neighbor map that represents the number of neighbor cells around a given cell in a radius of 0.22 mm with a color code that goes from purple (0–2 neighbors) to red (32–35 neighbors). Thus, each dot represents one m^+^ipRGC. Neighbor maps are better than isodensity maps for low density populations. N, nasal; S, superior. **(E)** Electroretinographic waves and their retinal origin. Under scoptopic conditions, the electroretinogram records the pSTR generated from RGCs (blue cells in the retinal drawing), the rod response, generated by rod-bipolar cells (pink cells), and the mixed wave generated by photoreceptors (purple) and ON-center bipolar cells (orange). Under higher light intensities, cone bipolar cells (green) generate the photopic b-wave. These images are original and have been created following our protocols described in: Alarcón-Martínez et al. (2009); Ortín-Martínez et al. (2010); Galindo-Romero et al. (2013); Nadal-Nicolás et al. (2018); and Gallego-Ortega et al. (2020).

Topographically both functional subtypes have a complementary distribution (Figures 1A,B). While the density of Brn3a^+^RGCs is higher in the central retina above the optic nerve along the naso-temporal axis, m^+^ipRGCs are more abundant in the areas of lowest Brn3a^+^RGC density.

Because the density of RGCs differs so much from central to peripheral retina (Figures 1A,B) to avoid topographic bias when manually quantifying RGCs one should sample standard areas in the central, medial, and peripheral retina (Parrilla-Reverter et al., 2009; Galindo-Romero et al., 2011).

## Photoreceptors

Cones and rods are the classical photoreceptors that is photoreceptors with a role in vision, while ipRGCs, which are also photoreceptors, differ from cones and rods in that they send afferents to the brain, as RGCs do, and in that their function is non-visual, as mentioned above.

Phototransduction starts in the photoreceptor outer segments where the opsins are stored. The outer segments face the retinal pigmented epithelium, and thus can be imaged in flatmounts with the vitreal side down. Opsin immunodetection is often used to visualize photoreceptors: rhodopsin (rods), S-opsin (S-cones), and L/M-opsin (L/M-cones). For cones it is possible to quantify each of the outer segments in flatmounts and assess their total numbers (Figures 1C,D) in health (Ortín-Martínez et al., 2010) and disease (García-Ayuso et al., 2013; Ortín-Martínez et al., 2015; Vidal-Sanz et al., 2015).

In rats, S-cones, which sense blue light, are more abundant in albino (41,028 ± 5,074; SD strain) than in pigmented animals (27,316 ± 2,235 PVG strain). However, the population of L/M cones, which perceive red-green light, is similar among them (albino: 231,736 ± 14,517; pigmented: 239,939 ± 6,494). S-cones are more abundant in the ventral retina and the extreme periphery, the retinal rims (Figure 1C) while the distribution of L/M cones resembles the topography of RGCs (compare panels **A** and **D** in Figure 1). Some cones express both opsins, the so-called dual cones. In both strains dual cones are ~8,000 per retina, and they are mainly found in the retinal rims (Ortín-Martínez et al., 2010).

Rods are very abundant, with an estimated population of 50 million (Nadal-Nicolás et al., 2018). They appear to be homogeneously distributed in the retina, as their outer segments are very dense (García-Ayuso et al., 2013) impeding the discrimination of individual ones, and precluding their automated quantification in flatmounts. Consequently, rod degeneration and neuroprotection are assessed in retinal cross-sections by quantifying the nuclei rows in the outer nuclear layer (Di Pierdomenico et al., 2018).

## Function

Since the early days of Gotch in 1903, who was the first to record the eye’s response to a flash of light in the frog retina, the electroretinogram (ERG) has been used as a non-invasive technique to assess retinal functionality and to establish more solid diagnoses of retinal pathologies. Numerous studies have characterized the functional response of the rat retina with full-field ERG (Alarcón-Martínez et al., 2009; Gallego-Ortega et al., 2020, 2021).

Depending on the type and intensity of the stimulus specific waves are obtained from specific retinal populations (Figure 1E). Very weak light stimuli under scotopic conditions, close to the rod threshold, raises the scotopic threshold response (STR), a slow negative corneal potential. This wave is formed by a positive (pSTR) and a negative (nSTR) component. The STR originates in the innermost part of the retina, where the RGC somas are located (Sieving et al., 1986; Frishman and Steinberg, 1989a, b). Although part of this wave is generated by amacrine cells (Naarendorp and Frumkest, 1991; Saszik et al., 2002), especially the positive component, many studies use the pSTR to assess the functional status of RGCs in albino and pigmented rats (Nadal-Nicolás et al., 2018; Boia et al., 2020; Gallego-Ortega et al., 2020, 2021) and mice (Valiente-Soriano et al., 2019; Norte-Muñoz et al., 2021). In adult rats the pSTR does not exceed 50 microvolts and can be generated with approximately −4.6 to −4 log cd.s/m^2^ light pulses.

In response to low-intensity full-field stimuli (intensities between −4 and −2 cd.s/m^2^ before the a-wave appears) the retina produces an electrical signal known as the rod response, consisting of a positive deflection of the electrophysiological tracing of about 250 μV amplitude. This response is generated by the rod bipolar cells, because of the decrease in glutamate release by rods and its action on mGluR6 receptors.

By increasing light intensity, the mixed response the best known of all waves, is obtained. The mixed response is the major component of the ERG and is used both clinically and experimentally. It consists mainly of two components: (i) the first to appear is a negative component, the a-wave, generated by the absorption of light in the photoreceptor outer segments and the closure of cGMP-gated cationic channels (Penn and Hagins, 1969; Sillman et al., 1969). In the adult rat the a-wave reaches 200 μV; and (ii) the a-wave is followed by a positive component, called the b-wave. There has been much controversy and theories about the origin of this ERG component, such as the hypothesis that it originates from Müller cells but nowadays everything points to it being generated exclusively by the ON-center bipolar cells (Masu et al., 1995; Green and Kapousta-Bruneau, 1999; Karwoski and Xu, 1999). At high light intensities (2 cd.s/m^2^), the rat b-wave reaches up to 800 microvolts in amplitude.

Under photopic conditions, with a background light of approximately 30 cd/m^2^ to eliminate the negative component generated by the hyperpolarization of the photoreceptors, the photopic b-wave emerges. This wave is generated exclusively by the cone pathway and is the result of the depolarization of the bipolar cone cells. In rat at intensities of 2 cd.s/m^2^ the photopic b-wave reaches peaks of ~150 microvolts.

There are other parameters that can be measured with full ERG such as the c-wave, d-wave, and oscillatory potentials, although these are seldom used.

There is some debate regarding retinal function in albino and pigmented rats. Some works describe a lower functional response in albino than pigmented rats (Nadal-Nicolás et al., 2018), while others do not observe a significant difference (Alarcón-Martínez et al., 2009).

## Discussion

The rat retina is widely used as a model of central nervous degeneration as well as to study specific ophthalmological diseases. As we have summarized here, its anatomy is well known and at present there are approaches to objectively assess the topography, number, and function of RGCs and photoreceptors, the retinal neurons most sensitive to injury. These automated tools have allowed to determine the fate of RGCs and cone photoreceptors in a variety of degeneration models, as well as their response to neuroprotective therapies (Hernandez et al., 2008; García-Ayuso et al., 2013; Ortín-Martínez et al., 2015; Vidal-Sanz et al., 2015; Millán-Rivero et al., 2018; Boia et al., 2022).

We have described the retina of 2-month-old female rats. Female rats are generally preferred over males because they are smaller and easier to handle. At 2 months of age, rats are young adults and most labs use animals within 2–4 months of age. However, it is important to have in mind that as the rat grows, so does the retina and although the total number of neurons does not change their density does. Retinal size stabilizes around 4–6 months of age and only in very old rats (22 months) there is loss of rods or cones but no of RGCs. Functionally, the highest wave amplitudes are obtained from 2-month-old rats thereafter all amplitudes decrease gradually (Nadal-Nicolás et al., 2018). Therefore, within the same experiment rats should have similar ages.

We show here that is feasible to study vision-forming (Brn3a^+^) and non-vision forming (melanopsin^+^) RGCs, and S- and L/M- cones in the same retina. Analyzing all these populations together is a very powerful approach hardly exploited and very useful to work with models that proceed with the death of all these populations, such as ischemia/reperfusion or retinal dystrophies or to assess the different vulnerability of each population to injury. For instance, m^+^ipRGCs are more resilient to injury or disease than Brn3a^+^RGCs (Nadal-Nicolás et al., 2015; Valiente-Soriano et al., 2015; Vidal-Sanz et al., 2017) and likewise, the susceptibility of L/M- and S- cones to the same insult differs (Hadj-Saïd et al., 2016; García-Ayuso et al., 2018; Di Pierdomenico et al., 2020).

Brn3a^+^RGCs and L/M-cones have their highest densities in the dorso-central retina in opposition to S-cones and m^+^ipRGCs that are densest in the ventral and dorsal periphery, respectively. The area of higher RGC and L/M cone density forms the visual streak (Salinas-Navarro et al., 2009; Ortín-Martínez et al., 2010), the region of the retina specialized to provide the best vision at some point in the visual space located along the naso-termporal axis above the optic nerve. The main characteristic of the visual streak is that it contains a high concentration of RGCs, L-cones, and cone bipolar cells.

Rats are foraging animals as well as prey. Thus, they do not need a central area of high resolution as primates, some birds, and reptiles have, but rather a good vision in the naso-temporal axis in a position that allows the animal to search for food without tilting its head. Furthermore, the high density of blue light-detecting cones in the ventral retina may be a key feature to detect birds of prey, their natural hunters.

In conclusion, although rats are nocturnal animals and their retinas do not have all anatomical features that human retinas have, such as the fovea, they are nonetheless a very good model for preclinical research. Besides the practical advantages of housing and relatively quick and easy breeding and development, there are many established models of ophthalmic disease and neuronal injury and, more importantly, as reviewed here, we have a very precise understanding of their retinal anatomy and function.

## Author Contributions

CG-R and MN-M: conceptualization, bibliographic search, and graphics. AG-O, KR-R, FL-R, and MG-R: conceptualization and bibliographic search. MV-S: conception and funding. MA-B: conception, funding, writing the first draft of the manuscript. All authors: revising the article critically for important intellectual content. All authors contributed to the article and approved the submitted version.

## Funding

This research was funded by the Spanish Ministry of Economy and Competitiveness (project: PID2019-106498GB-I00 (MV-S), by the Instituto de Salud Carlos III, Fondo Europeo de Desarrollo Regional “Una manera de hacer Europa” project: PI19/00071 (MA-B), the RETICS subprograms of Spanish Networks OftaRed RD16/0008/0026 and RD16/0008/0016 (MV-S).
